# Robust Stitching Interface and Deep Learning Empowered Hydrogel Human‐Machine Interface

**DOI:** 10.1002/advs.77052

**Published:** 2026-08-06

**Authors:** Hao Dong, Yiming Luo, Chuanliu Liu, Mahamood Modupe Rasheedat, Wenjiayi Tan, Fangyu Zhao, Yixue Zhang, Chris Connor, Hua Hou, Hamdy Khamees Thabet, Ben Bin Xu, Huige Wei

**Affiliations:** ^1^ State Key Laboratory of Bio‐based Fiber Materials Tianjin Key Laboratory of Multivariate Identification for Port Hazardous Chemical Substances Tianjin Key Laboratory of Brine Chemical Engineering and Resource Eco‐Utilization College of Chemical Engineering and Materials Science Tianjin University of Science and Technology Tianjin China; ^2^ School of Engineering Physics and Mathematics Northumbria University Newcastle Upon Tyne UK; ^3^ College of Chemistry Fuzhou University Fuzhou China; ^4^ School of Materials Science and Engineering Taiyuan University of Science and Technology Taiyuan China; ^5^ Center for Scientific Research and Entrepreneurship Northern Border University Arar Saudi Arabia

**Keywords:** deep learning, human‐computer interaction, information transmission, interface functionalization

## Abstract

Mechanical mismatch and weak interfacial adhesion between hydrogels and encapsulation layers, particularly polyethylene terephthalate (PET), remain the primary cause of signal distortion in dynamic sensing applications. We address this through a molecular design strategy that exploits synergistic carboxylate anion–quaternary ammonium interactions to simultaneously strengthen the hydrogel–PET interface and the bulk hydrogel network. The strategy operates through two coupled mechanisms. Fe^3^^+^ ions bridge the –COO^−^ groups of sodium acrylate with the electronegative –C═O groups of PET, converting a mechanically mismatched contact into a chemically bonded interface that suppresses interfacial slippage under dynamic loading. Concurrently, electrostatic cross‐linking within the polyacrylamide network reinforces bulk mechanical integrity. The resulting MA_2.5_D_1_GF hydrogel delivers a 9.2‐fold increase in adhesion strength to PET and a 2.6‐fold improvement in tensile stress at break relative to the unfunctionalized control—demonstrating that interfacial and bulk reinforcement are achieved within the same molecular architecture rather than as competing trade‐offs. Integrated with deep learning signal processing, the stable hydrogel–PET interface enables consistent signal acquisition and reliable human‐machine interaction under prolonged dynamic operation—establishing a molecularly programmable adhesion strategy with direct applicability to smart interface and wearable sensing platforms.

## Graphical abstract

A molecular design strategy is presented that exploits synergistic carboxylate anion–quaternary ammonium interactions to simultaneously strengthen the hydrogel–PET interface and the bulk hydrogel network. Integrated with deep learning signal processing, the stable hydrogel–PET interface enables consistent signal acquisition and reliable human‐machine interaction under prolonged dynamic operation.

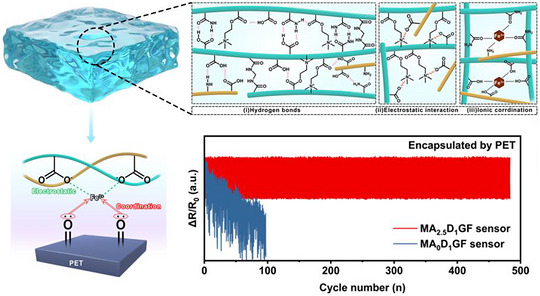

## 1 Introduction

With the rapid rollout of flexible electronics in various applications such as health monitoring [1], soft robotics [2], and implantable biosensing [3, 4], hydrogels have emerged as a promising component for future flexible sensors due to their high water content, biocompatibility, and mechanical flexibility [5, 6, 7]. Yet this same hydrophilicity renders them intrinsically vulnerable to dehydration, swelling, and chemical degradation in open environments, directly undermining the long‐term stability that real‐world deployment demands [8, 9]. The elastic encapsulation layers such as polydimethylsiloxane (PDMS) [10], thermoplastic polyurethanes (TPU) [11], ethylene vinyl acetate (EVA) [12], and polyethylene terephthalate (PET) [13] can address this by imposing a physical barrier against humidity, temperature, and chemical interference [14, 15]. Among these, PET has emerged as the encapsulant of choice, combining superior barrier performance, chemical inertness, and optical transparency in a single, processable material.

This apparent solution, however, introduces a structural liability. The significant mechanical mismatch and weak interfacial interactions between the hydrogel network and PET generate stress localization under deformation [16], driving relative interlayer sliding. The consequences cascade through the sensing unit: structural integrity is compromised, and the electrical response becomes corrupted by nonlinear signal drift, response hysteresis, and outright distortion—degrading both measurement accuracy and operational lifespan [17, 18]. Strong encapsulation protection and reliable dynamic sensing are therefore in direct conflict within current hydrogel–PET architectures, and resolving this interfacial incompatibility is the prerequisite for deploying hydrogel sensors in demanding, long‐duration applications.

Existing strategies to strengthen hydrogel–PET interfacial bonding include adhesive coating [19, 20], surface modification of the encapsulation layer [21], or grafting functional groups onto hydrogel surfaces [22, 23]. Adhesive coatings increase interfacial impedance and compromise flexibility; surface modification and grafting approaches suffer from process complexity and poor durability of the modified interface. While some reported hydrogels [24] can effectively mitigate interface slippage and delamination during sensor operation, challenges remain to be tackled in simultaneously achieving robust mechanical strength and stable sensing performance, despite strong interfacial bonding [25]. This conflict has two distinct mechanistic origins. First, incorporating polar groups to enhance adhesion disrupts cross‐linking uniformity within the hydrogel network, directly reducing tensile strength. Second, some adhesive components (i.e., acrylic acid [26], polydopamine [27], polysaccharides [28]) undergo non‐specific adsorption with conductive fillers, corrupting charge transfer pathways and manifesting as reduced sensitivity or elevated signal noise [29, 30, 31, 32]. Therefore, developing a hydrogel architecture that resolves this trade‐off is both a critical unmet need and the central challenge this work addresses.

**FIGURE 1:**
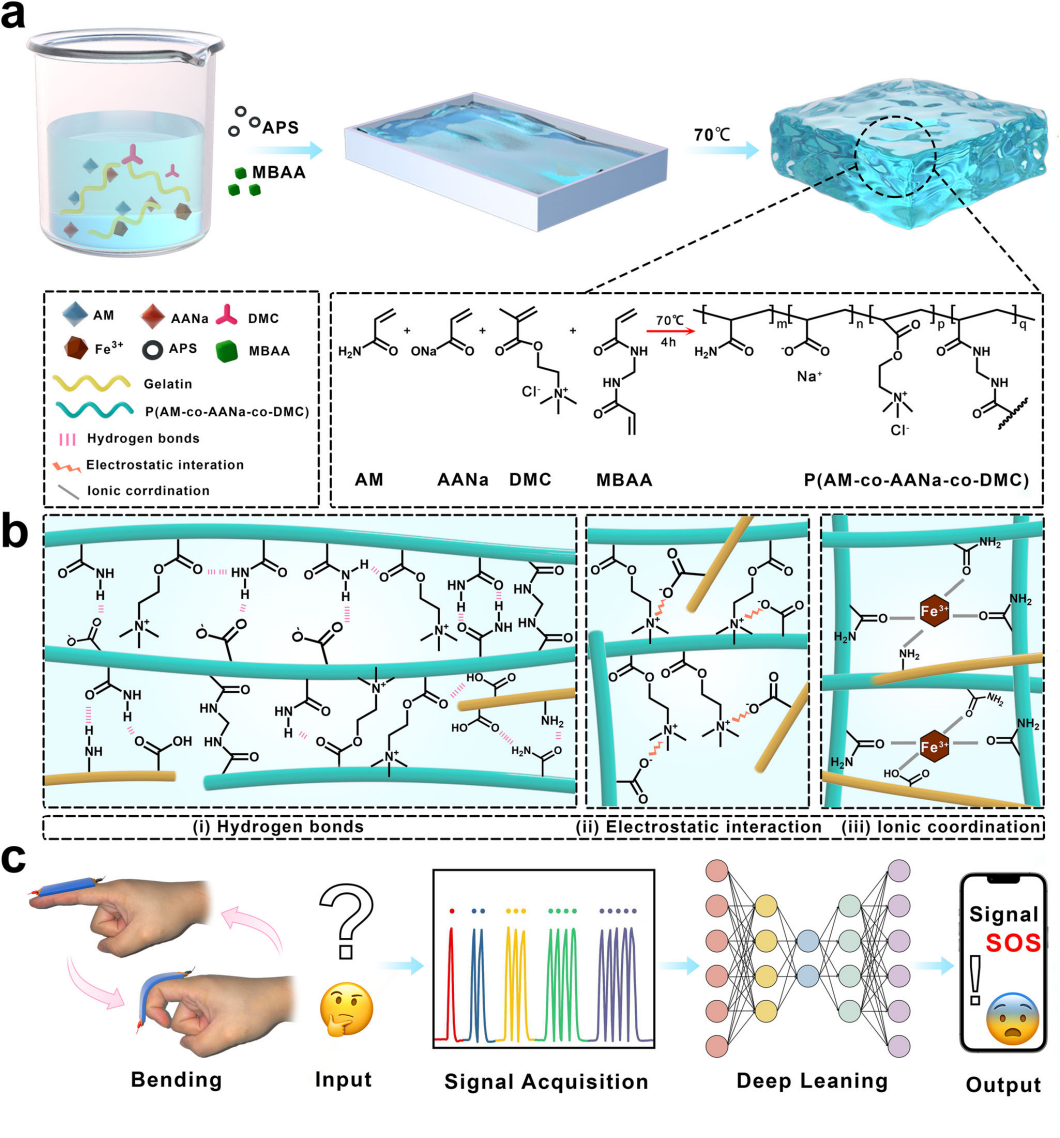
The molecular design and fabrication of MA*_x_* D*_y_* GF hydrogels (a) flow chart of preparation. (b) Internal cross‐linking structure. (c) Schematic of deep learning‐assisted information processing and transmission.

In this work, we present MA_2.5_D_1_GF, a polyacrylamide‐based hydrogel architecture that resolves the three‐way trade‐off between mechanical strength, interfacial adhesion, and sensing performance through a single molecular design strategy—synergistic interaction between carboxylate anions and quaternary ammonium salt groups (Figure 1). The incorporation of sodium acrylate (AANa) as a functional monomer confers the following significant advantages to the MA_2.5_D_1_GF hydrogel: (1) Sodium acrylate forms dynamic electrostatic cross‐links within the network, enhancing structural cohesion and significantly improving tensile strain, tensile stress, and toughness; (2) Sodium acrylate are bridged with PET by iron ions, significantly enhancing the hydrogel's adhesion strength and thereby improving the overall structural stability of the sensor; (3) The zwitterionic effect induced by sodium acrylate optimizes the internal ion distribution of the hydrogel, leading to enhanced sensing sensitivity and signal stability. As a proof of concept, MA_2.5_D_1_GF is integrated into an adhesion‐enhanced intelligent sensing system for human‐machine interaction. Coupled with deep learning signal processing, the system achieves high‐reliability information transmission under prolonged dynamic operation — demonstrating a molecularly programmable design route toward wearable flexible electronics that no longer treat adhesion, mechanics, and sensing as competing objectives.

## 2 Results and Discussion

### 2.1 Design Strategy of MA*_x_* D*_y_* GF Hydrogels for Deep Learning‐Assisted Information Processing and Transmission

The preparation route of the MA*_x_* D*_y_* GF hydrogels is illustrated in Figure 1a, where M, A, D, G, and F stand for acrylamide (AM), sodium acrylate (AANa), ethacryloyloxyethyltrimethylammonium chloride (DMC), gelatin, and ferric sulfate (Fe_2_(SO_4_)_3_), respectively. Gelatin is a natural polymer bearing rich polar groups in molecular chains and plays a vital role in tuning hydrogel performance. The variables *x* and *y* denote the mass of AANa and DMC, respectively, while the mass of AM is kept constant (Table S1). The incorporation of AANa significantly increases the number of carboxylate groups (‐COO^−^) within the system, while the ‐COO^−^ engage in electrostatic interactions with the quaternary ammonium groups (‐N^+^(CH_3_)_3_) of DMC molecules.

This synergistic effect substantially strengthens the cohesive strength of the hydrogel network [33]. In addition, the Fe^3+^ added to the system can form coordination bonds with ‐COO^−^. The hydrogel network composed of hydrogen bonds, electrostatic interactions, and coordination bonds effectively enhances the mechanical properties of the hydrogel (Figure 1b) [34]. With the help of deep learning algorithms, an adhesion‐enhanced intelligent sensing system for high‐efficiency human‐machine interactions is developed (Figure 1c). By constructing a custom gesture encoding library, users can trigger the system interface to display corresponding information in real time through distinct finger flexion movements, enabling wireless translation of physical gestures into digital commands and therefore significantly enhancing interaction efficiency.

### 2.2 Characterization of MA*_x_* D*_y_* GF Hydrogels

**FIGURE 2:**
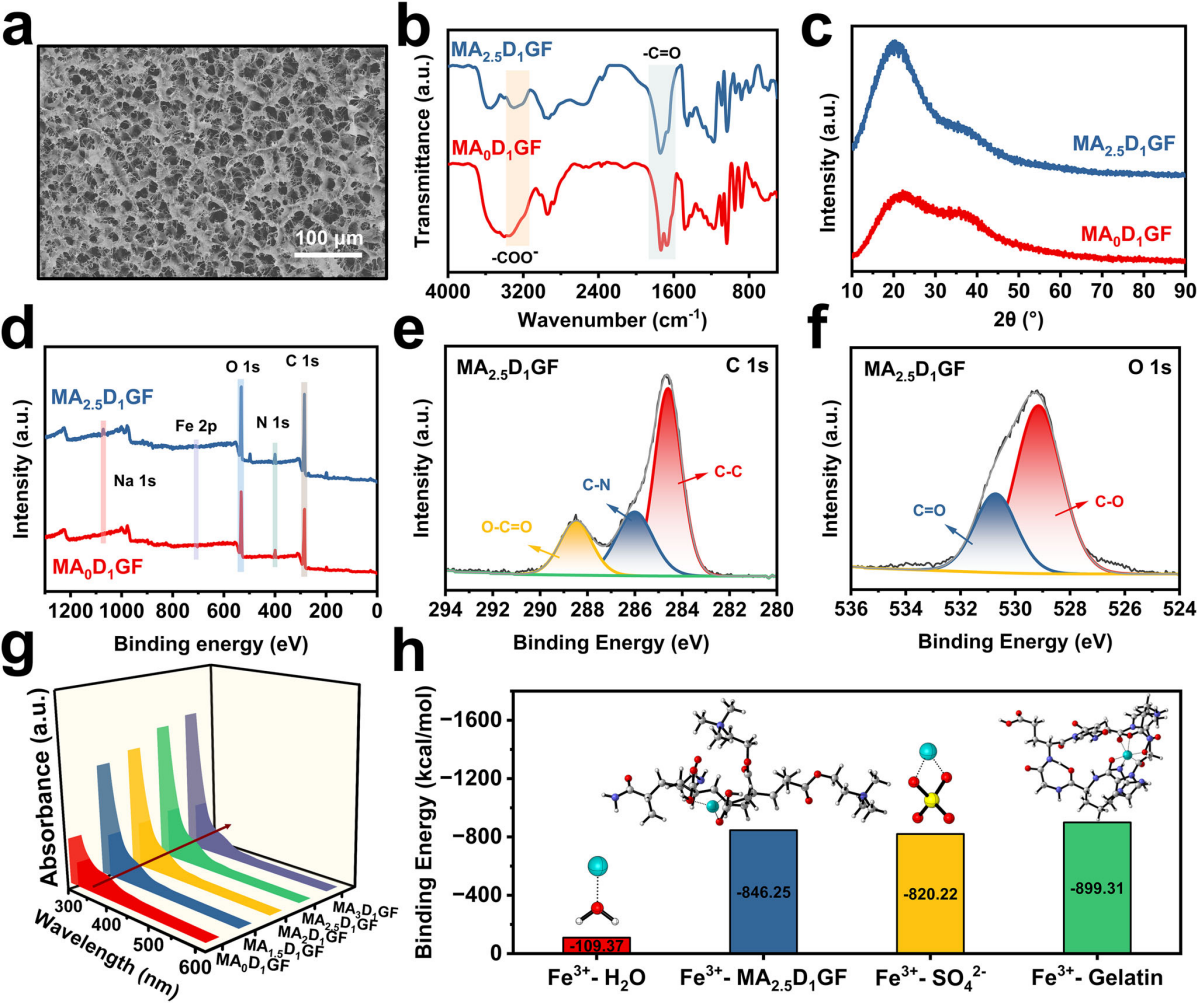
Characterizations of the MA*_x_* D*_y_* GF hydrogels. (a) SEM image of the MA_2.5_D_1_GF hydrogel. (b) FTIR spectrum of the MA*_x_* D*_y_* GF hydrogels. (c) XRD patterns of MA_0_D_1_GF and MA_2.5_D_1_GF hydrogels. (d) XPS spectra of MA_0_D_1_GF and MA_2.5_D_1_GF hydrogels. XPS spectra of (e) C and (f) O elements in the MA_2.5_D_1_GF hydrogel. (g) UV–vis spectrum of the MA*_x_* D*_y_* GF hydrogel. (h) The binding energy between Fe^3+^‐H_2_O, Fe^3+^‐MA_2.5_D_1_GF, Fe^3+^‐SO_4_^2−^, and Fe^3+^‐Gelatin.

The MA_2.5_D_1_GF hydrogel forms a continuous, homogeneous three‐dimensional network in which densely packed cross‐linking nodes — established through hydrogen bonding, electrostatic interactions, and metal coordination — are interwoven with flexible chain segments, producing a well‐interconnected reticular microarchitecture (Figure 2a). The Fourier transform infrared spectroscopy (FTIR, Figure 2b) confirms a characteristic ‐COO^−^ vibration peak at 3300 cm^−1^ and a significantly enhanced C═O stretching vibration at 1750 cm^−1^. Both spectral features originate from incorporated AANa groups when compared with the control sample lacking AANa (MA_0_D_1_GF), collectively demonstrating successful integration into the hydrogel network [35, 36]. The x‐ray diffraction (XRD) patterns (Figure 2c) unveil an enhanced diffraction peak intensity for the MA_2.5_D_1_GF hydrogel, attributed to carboxylate anion interactions with amide bonds and amino groups that regulate chain arrangement and improve crystallization [24, 37].

X‐ray photoelectron spectroscopy (XPS, Figure 2d and Figure S1) clearly signals a distinct Na 1s peak at 1070 eV in the full spectrum, which confirms the presence of sodium. High‐resolution C 1s and O 1s spectra (Figure 2e,f) reveal peaks at 284.6 eV (C–C), 286.1 eV (C–N), and 288.4 eV (O–C═O), as well as at 529.1 eV (C–O) and 530.7 eV (C═O) [38]. Notably, these peaks exhibit significant binding energy shifts compared to those of MA_0_D_1_GF (Figures S2 and S3), further substantiating the chemical integration of AANa into the polymer network.

The ultraviolet–visible (UV–vis) absorption spectroscopy (Figure 2g) confirms Fe^3^^+^– carboxylate coordination through a pronounced spectral shift upon AANa introduction [39]. The density functional theory (DFT) calculations were performed using Gaussian 09 software. The interaction strength was evaluated by comparing the binding energies (*E*) of Fe^3+^ with various ligand models, with lower binding energy values indicating stronger interactions between the metal ion and the ligands [40]. As shown in Figure 2h, the binding energies *E*(Fe^3+^‐Gelatin) and *E*(Fe^3+^‐MA_2.5_D_1_GF) of Fe^3+^ with gelatin and MA_2.5_D_1_GF, respectively, are significantly lower than its binding energy *E*(Fe^3+^‐H_2_O) with water molecules, indicating that Fe^3+^ is more inclined to form coordination bonds with the carboxyl groups (‐COOH) on the gelatin chains and the carboxylate ions (‐COO^−^) in MA_2.5_D_1_GF rather than coordinating with water molecules [41]. The theoretical calculation results agree with the spectral results, jointly establishing the specific Fe^3^^+^–polymer coordination mechanism that underpins the network's cross‐linking architecture.

### 2.3 Mechanical and Adhesive Properties of MA*_x_* D*_y_* GF Hydrogels

**FIGURE 3:**
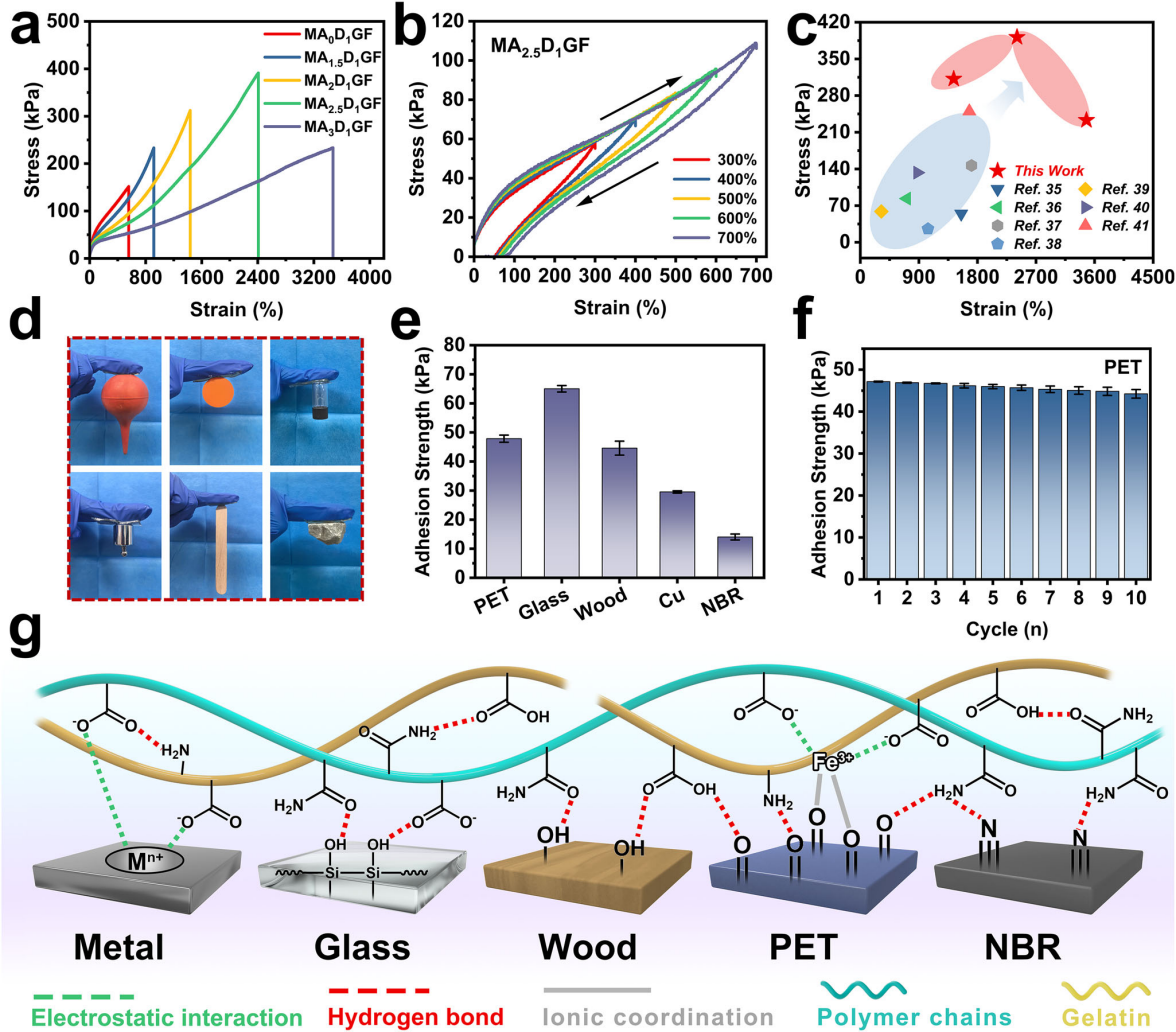
Mechanical and adhesion properties of hydrogels. (a) Typical tensile stress–strain curves of MA*_x_* D*_y_* GF hydrogels. (b) Tensile stress–strain curves of the MA_2.5_D_1_GF hydrogel at different strains (300%, 400%, 500%, 600%, and 700%). (c) Comparison of mechanical properties of MA*_x_* D*_y_* GF hydrogels with reported hydrogels (Ref [52], Ref [53], Ref [54], Ref [55], Ref [56], Ref [57], and Ref [58]). (d) Digital photographs showing strong adhesion of MA_2.5_D_1_GF hydrogel to different substrates of rubber, plastic, glass, metal, wood, and stone, and (e) the adhesion strength of MA_2.5_D_1_GF hydrogel to PET, glass, wood, Cu, and NBR (error bars show mean ± SD, *n* = 3). (f) Adhesion strength of the MA_2.5_D_1_GF hydrogel to PET after 10 cycles of cyclic peeling (error bars show mean ± SD, *n* = 3). (g) Mechanism of the MA_2.5_D_1_GF hydrogel adhesion to different substrates.

The multiple cross‐linking network — spanning hydrogen bonds, electrostatic interactions, and coordination bonds — translates directly into exceptional mechanical performance [42, 43, 44, 45, 46]. The MA_2.5_D_1_GF hydrogel achieves a tensile strain of 2409%, a tensile stress of 391.3 kPa, and a toughness of 4.2 MJ·m^−3^, which are 4.3, 2.6, and 8.1 times higher, respectively, over the AANa‐free control MA_0_D_1_GF hydrogel (Figure 3a, Figure S4 and Video S1). In addition, the tensile strain of the MA_2.5_D_1_GF hydrogel further increases with the addition of AANa, reaching a maximum strain of 3475% at an AANa:DMC mass ratio of 3:1.; meanwhile, the hydrogel demonstrates excellent resilience under multiple different strain conditions (Figure 3b). The hydrogel maintained good mechanical stability even after 500 cycles over a period of approximately 30 days (Figure S5). Compared with the reported hydrogels (Figure 3c and Table S2), the MA_2.5_D_1_GF hydrogel in this study shows significant advantages in mechanical properties, further confirming its outstanding comprehensive mechanical performance.

Strong, durable interfacial adhesion is a prerequisite for signal accuracy and stability in flexible sensors [47, 48]. The MA_2.5_D_1_GF hydrogel can achieve tight adhesion to a variety of substrates (rubber, plastic, glass, metal, wood, and stone) (Figure 3d). A 1 × 4 cm sample bonded between two glass plates sustained a continuous static load of 14.7 N over a two‐week period without interfacial failure (Figure S6), confirming that adhesion is structurally robust.

The adhesion performance of MA*_x_* D*_y_* GF hydrogels was further evaluated using lap shear tests (Figure S7) [49]. The testing reveals that adhesion strength increases then decreases with AANa content, with MA_2.5_D_1_GF at the optimum — delivering a 9.2‐fold increase in adhesion strength to PET relative to the AANa‐free control group (Figure S8). The enhanced adhesion is attributed to Fe^3+^ ions coordinating simultaneously with both the ‐COO^−^ group and the negatively charged sites of PET, thereby forming a bridging effect (Figure S9) [50, 51]. Considering both mechanical and adhesive properties comprehensively, the MA_2.5_D_1_GF hydrogel was ultimately selected for further investigation, which has impressive adhesion strengths on PET, glass, wood, Cu, and NBR (nitrile butadiene rubber) surfaces as 47.8 ± 1.2, 65.1 ± 1.1, 44.6 ± 2.4, 29.5 ± 0.4, and 14.1 ± 1.1 kPa, respectively (Figure 3e). High adhesion resolves the challenging issue of weak bonding between hydrogels and PET films, providing crucial support for the practical application of hydrogel sensors.

We next assessed the long‐term adhesion tests after bonding to the substrate surfaces for 30 days; the adhesion performance on PET was maintained or enhanced; adhesion to glass, wood, Cu, and NBR showed only a slight decrease (Figure S10). Over ten repeated adhesion‐peeling cycles (Figure 3f and Figures S11–S14) across multiple substrates, performance degradation was negligible — confirming that the multi‐mechanism adhesion architecture is reversible without fatigue. Specifically, NBR rubber possesses excellent chemical compatibility and mechanical matching with the hydrogel. Cyclic loading facilitates the simultaneous enhancement of interfacial adhesion and the optimization of bulk cohesive performance, which ultimately leads to an evolution of adhesion strength characterized by an initial decrease followed by an increase. This robust and reliable adhesion capability primarily arises from multiple mechanisms, including electrostatic interactions, hydrogen bonding, and physical entanglement, enabled by abundant functional groups such as carboxyl and hydroxyl groups within the MA_2.5_D_1_GF hydrogel network (Figure 3g) [33, 59]. Benchmarked against reported hydrogel materials (Table S3), MA_2.5_D_1_GF simultaneously leads in both strain and adhesion performance, establishing it as a uniquely capable adhesive layer for demanding flexible sensing interfaces.

### 2.4 The Evaluation of Sensing Performance

**FIGURE 4:**
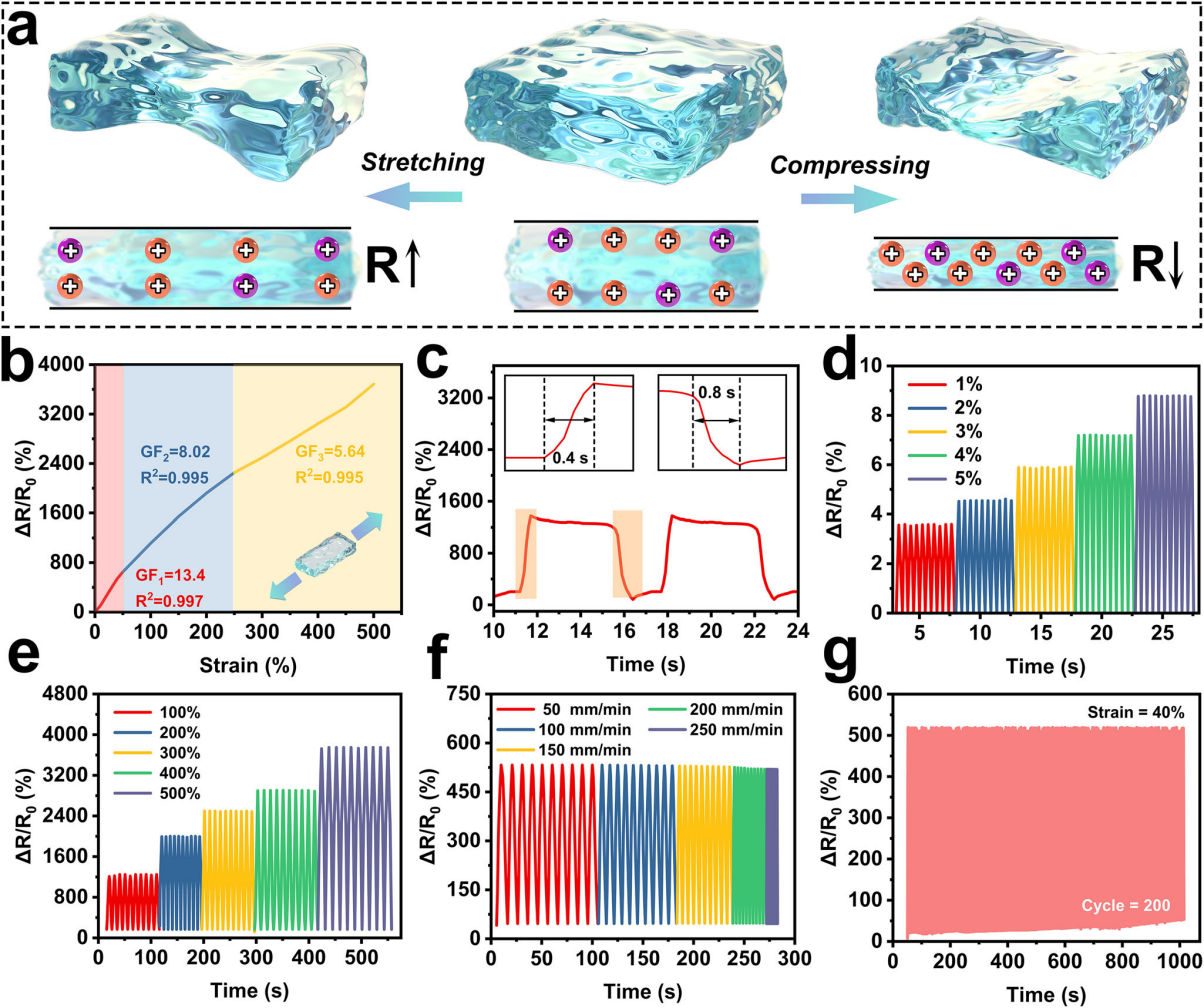
The assessment of sensing performance. (a) Schematic of mechano‐sensing under deformation. (b) Gauge factor (*GF*) of hydrogel sensor under various tensile strains (0%–500%). (c) Response and relaxation times of hydrogel sensors. (d) Relative resistance changes under different strains (1%–5%). (e) Relative resistance changes under different strains (100%–500%). (f) Changes in the relative resistance at different stretching rates (50–150 mm min^−1^) under a fixed strain of 40%. (g) Changes in the relative resistance of the hydrogel sensor after 200 stretches at a fixed strain of 40%.

In ion‐conductive hydrogels, resistance is governed by two coupled parameters: ion transport channel connectivity and volumetric ion concentration [60]. Tension and compression perturb both parameters in opposing directions, producing predictably opposite resistance responses (Figure 4a). Under tension, the polymer network elongates, pores narrow, and ion transport pathways become more tortuous — simultaneously diluting volumetric ion concentration and increasing migration collision probability, collectively reducing ionic conductivity. Under compression, pore compaction shortens ion migration paths and enhances channel connectivity, while volumetric ion concentration increases — together improving ionic conductivity. This mechanistically transparent structure–property relationship underpins the predictable, strain‐dependent sensing behavior of MA_2.5_D_1_GF.

The 1% – 500% strain range was divided into three intervals (1% – 50%, 50% – 250%, 250% – 500%) for piecewise linear fitting. The relative resistance (Δ*R*/*R*_0_) of MA_2.5_D_1_GF hydrogel sensor (Figure 4b) exhibits a multi‐stage linear relationship with strain, with gauge factors (*GF* = (Δ*R/R*_0_)/*ε*) of 13.40 (0%–50%), 8.02 (50%–250%), and 5.64 (250%–500%) — reflecting the progressive evolution of network geometry and ion transport architecture across the full deformation range. As shown in Figure 4c, the sensor's response and relaxation times at 100% strain are 0.4 and 0.8 s, respectively, confirming rapid dynamic sensing capability sufficient for real‐time human motion capture.

MA_2.5_D_1_GF resolves ΔR/R_0_ signals continuously from low strain (1%–5%) to high strain (100%–500%), maintaining a high signal‐to‐noise ratio and excellent repeatability across cyclic loading — a detection range that spans both subtle physiological signals and large‐amplitude body movements within a single sensing platform (Figure 4d,e). Output signals remain highly consistent across stretching rates from 50 to 150 mm min^−^^1^ at 40% strain, confirming rate‐independent sensing response (Figure 4f). After 200 consecutive stretch‐release cycles at 40% strain, resistance signals show no measurable attenuation or drift (Figure 4g), establishing the durability and reliability required for sustained wearable device operation.

The MA_2.5_D_1_GF hydrogel demonstrates well‐defined pressure sensing performance across a wide operational range. Sensitivity (*S* = (*ΔR/R*_0_)/Δ*P*, Figure S15) follows a multi‐stage linear response: *S*_1_ = 0.051 kPa^−^^1^ (0–10 kPa), *S*_2_ = 0.0048 kPa^−^^1^ (10–20 kPa), and *S*_3_ = 0.0036 kPa^−^^1^ (20–70 kPa), reflecting the progressive compaction of hydrogel network and corresponding evolution of ion transport channel geometry across full pressure range. The sensor accurately discriminates between discrete pressure magnitudes during cyclic compression (Figures S16 and S17), with output resistance signals closely tracking applied pressure waveforms. Under ten cycles at varying pressures (2–10 kPa, Figure S18), waveform peaks remain synchronized and consistent, confirming both signal fidelity and inter‐cycle reproducibility. Sensing performance remains stable across frequencies from 0.3 to 2 Hz (Figure S19), establishing rate‐independent dynamic reliability. After 200 compression cycles at 0.5 kPa (Figure S20), no measurable signal attenuation appears, demonstrating fatigue resistance and long‐term pressure sensing durability. Benchmarked against reported hydrogel sensors (Table S4), MA_2.5_D_1_GF simultaneously achieves superior mechanical properties, high sensitivity, and a wide sensing range, establishing it as a reliable platform for flexible pressure sensing applications.

### 2.5 Demonstration of Motion Detection and Communication

**FIGURE 5:**
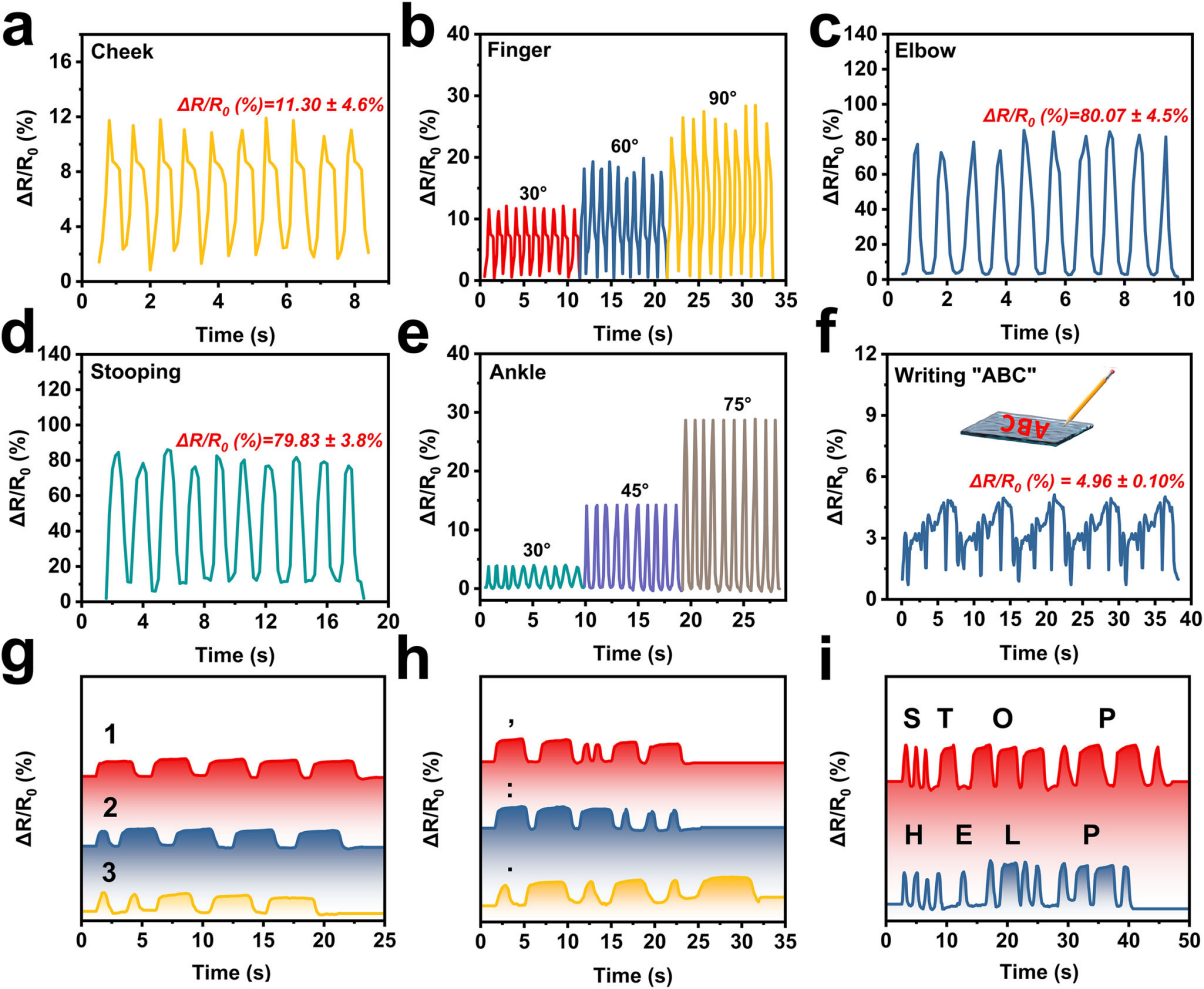
The MA_2.5_D_1_GF hydrogel sensors monitored relative changes in electrical resistance during human activities: (a) Cheek, (b) Finger, (c) Elbow, (d) Stooping, and (e) Ankle. (f) Handwriting recognition of ABC letters by the sensor. Sensor information transmission signals, including (g) numbers, (h) punctuation marks, and (i) words.

Real‐world sensing environments impose coupled tension and compression simultaneously [61] — a condition that isolated uniaxial testing cannot validate. To evaluate MA_2.5_D_1_GF under physiologically realistic combined loading, the sensor was mounted across multiple human joints and body segments, with resistance responses used to monitor a full spectrum of motion behaviors from subtle to large‐scale. At the subtle end of the detection range (Figure 5a), the sensor resolves cheek vibrations with a Δ*R/R*_0_ standard deviation of only 4.6% — confirming the signal stability required to extract low‐amplitude physiological signals from noise. At intermediate scale, resistance response tracks finger bending angle incrementally from 0° to 90° with clear, monotonic signal progression (Figure 5b). At large scale, the sensor monitors repetitive, high‐amplitude movements — elbow flexion‐extension, trunk bending, and leg lifting — with consistent, stable output across cycles (Figure 5c–e). This continuous performance across three orders of motion amplitude within a single device confirms the broadband mechanical sensing capability of the MA_2.5_D_1_GF architecture.

The PET‐encapsulated MA_2.5_D_1_GF sensors resolve individual character strokes (A, B, C) and continuous sequences (ABC) with near‐identical resistance change curves between repetitions (Figure 5f and Figure S21), demonstrating the signal reproducibility necessary for reliable handwritten information capture and recognition. Exploiting the sensor's angle‐discriminating capability, a Morse code encoding scheme was implemented (Figure S22) by defining 30° bending as “dot” and 90° bending as “dash” (Figure S23). The system accurately outputs numbers, punctuation, and symbols through combined bending sequences (Figure 5g,h), and successfully generates meaningful commands — including “STOP” and “HELP” (Figure 5i). The interfacial compatibility between MA_2.5_D_1_GF and the PET encapsulation layer directly translates into sensing durability: the encapsulated sensor maintains stable, reliable signal output across nearly 500 bending cycles without degradation. The AANa‐free MA_0_D_1_GF fails this test decisively with explicit performance degradation after just 20 cycles (Figure S24). The Fe^3^^+^‐mediated interfacial bonding architecture as the decisive structural factor governing long‐term sensing reliability, enabling assistive and wearable communication technologies.

### 2.6 Deep Learning Empowered Sensing Signal Processing and Transmission

**FIGURE 6:**
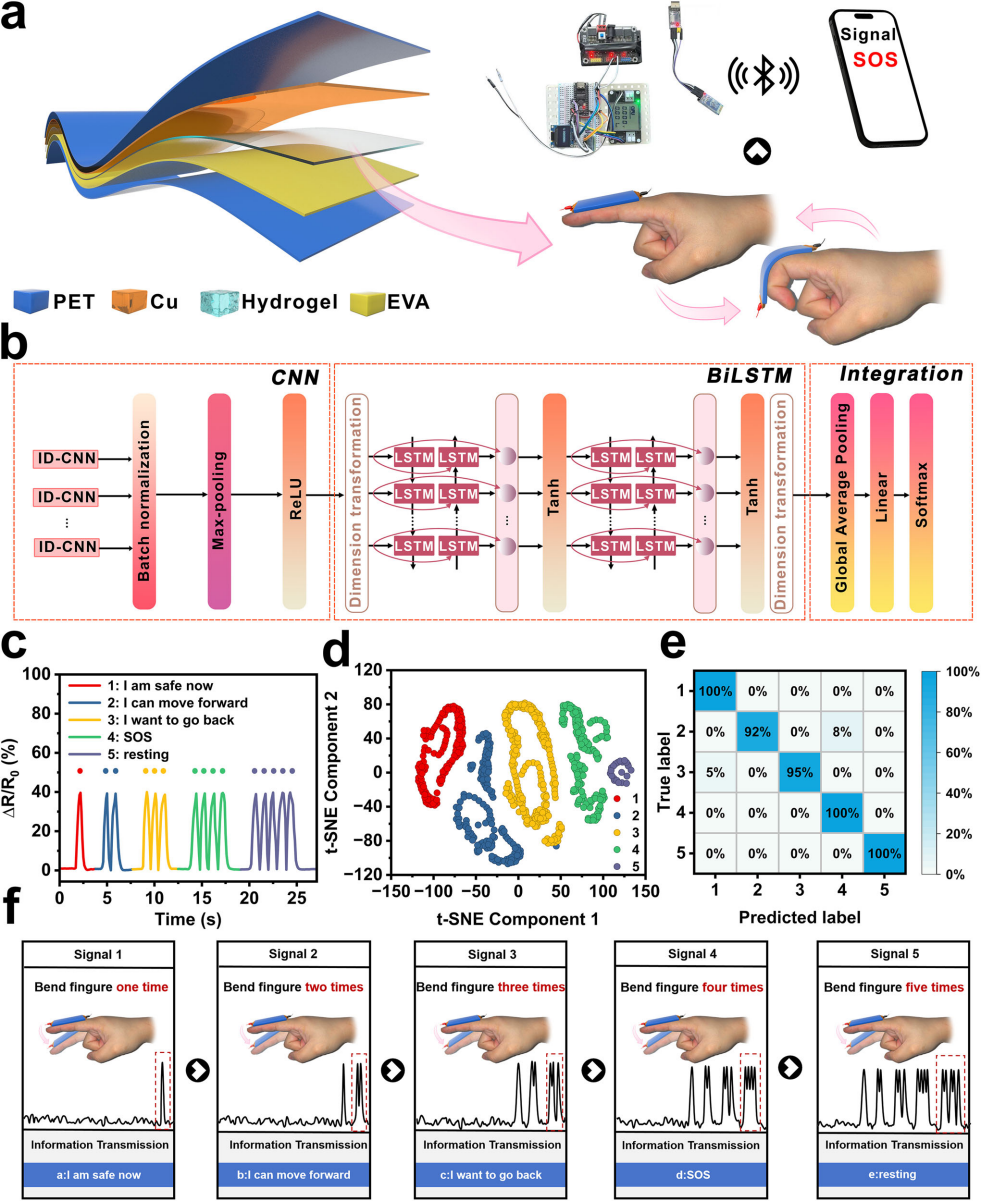
Deep learning empowered human‐machine interface. (a) Wireless information transmission based on the MA_2.5_D_1_GF hydrogel sensing system. (b) Deep learning algorithm process. (c) Signal. (d) t‐distributed stochastic neighbor embedding (t‐SNE) visualization graph. (e) Confusion matrix plot of recognition results. (f) Output statements of the assembled wireless information transmission system in different states.

Building on this stable sensing foundation, an intelligent sensing system was constructed by integrating the PET‐encapsulated MA_2.5_D_1_GF sensor with a custom deep learning recognition architecture and supporting hardware modules (Figure 6a) [62, 63, 64, 65]. The system's analytical core is a purpose‐designed CNN‐BiLSTM hybrid neural network (Figure 6b), whose architecture is explicitly optimized for the temporal and morphological complexity of hydrogel sensor signals. The front end comprises three convolutional modules — each with a 5 × 1 kernel — with channel depth progressively increasing from 16 to 32 to 64, systematically extracting high‐order local pattern features from raw signal waveforms at increasing levels of abstraction. A 128‐unit bidirectional long short‐term memory (BiLSTM) layer follows, capturing long‐range temporal dependencies and dynamic evolution patterns in both forward and reverse sequence directions. An attention mechanism is incorporated to focus model capacity dynamically on signal segments of highest discriminative value, suppressing noise interference and improving generalization — ensuring that recognition performance reflects genuine signal content rather than training set memorization.

The resulting system implements a five‐instruction Morse code protocol defined by peak count: one peak — “I am safe now”; two peaks — “I can move forward”; three peaks — “I want to go back”; four peaks — “SOS”; five peaks — “resting” (Figure 6c). t‐SNE dimensionality reduction of extracted features confirms that all five signal categories form clearly separable clusters in two‐dimensional space, demonstrating that the CNN‐BiLSTM architecture captures discriminatively distinct representations for each instruction class (Figure 6d). Trained on 1000 signal groups, the model converges within 50 epochs — loss stabilizing at 0.021 (Figure S25) and validation accuracy at 98.06% (Figure S26) — confirming both training efficiency and strong generalization. Confusion matrix analysis verifies high‐precision classification across all five categories with minimal inter‐class confusion (Figure 6e).

To validate end‐to‐end system performance under practical conditions, the PET‐encapsulated MA_2.5_D_1_GF sensor was mounted on a volunteer's finger. Predefined bending gestures generated strain signals that were captured, processed, and transmitted in real time, with classified instructions displayed live on a remote receiver (Figure 6f and Video S2). This demonstration confirms three system‐level capabilities simultaneously: signal capture fidelity under dynamic loading, low‐latency wireless transmission, and real‐time recognition, collectively endowing MA_2.5_D_1_GF as a viable sensing platform for wearable emergency communication and remote human‐machine interface.

## 3 Conclusions

This work describes a molecularly programmable design strategy — centred on AANa‐driven synergistic carboxylate anion–quaternary ammonium interactions — that resolves the three‐way trade‐off between interfacial adhesion, mechanical strength, and sensing performance that has constrained hydrogel‐based flexible sensors. The resulting MA_2.5_D_1_GF hydrogel delivers a 9.2‐fold increase in PET adhesion strength, a 2.6‐fold improvement in tensile stress, and a gauge factor of 13.40 in the 0%–50% strain range. Integrated with a purpose‐designed CNN‐BiLSTM deep learning architecture, the encapsulated sensing system achieves 98.06% classification accuracy and real‐time wireless signal transmission. Beyond this specific system, the Fe^3^^+^‐mediated bridging strategy and multi‐mechanism adhesion framework it establishes offer a transferable design principle for any application requiring mechanically robust, chemically bonded hydrogel–substrate interfaces. This positions MA_2.5_D_1_GF as a credible material platform for next‐generation wearable health monitoring, assistive communication, and human‐machine interaction.

## 4 Experimental Section

### 4.1 Materials

Acrylamide (99%, AM) and ferric sulfate (98%, Fe_2_(SO_4_)_3_) were purchased from Tianjin Jindong Tianzheng Fine Chemical Reagent Factory. Methacryloyloxyethyltrimethylammonium chloride (75%, DMC), sodium acrylate (99.5%, AA), and gelatin (99%) were purchased from Macklin (P. R. China). N,N'‐methylenebisacrylamide (99%, MBAA) and ammonium persulfate (>99%, APS) were purchased from Biddell Pharmaceuticals. Deionized water (DI) was homemade in the laboratory. All reagents were used as received, unless otherwise stated. Ethical approval for this work was not required. Participants took part with written informed consent.

### 4.2 Characterizations

The top view of the freeze‐dried samples was observed using a field emission high‐resolution scanning electron microscope (FE‐SEM) model Apreo from FEI, USA. The samples were prepared by mixing potassium bromide with different components of the gel powder in a ratio of about 100:1 and then pressed into tablets, and the hydrogels were scanned using a VECTOR 22 infrared spectrometer from Bruker, Germany, at a resolution of 4 px^−1^ in a scanning interval of 400–4000 cm^−1^. The crystal structure information of the hydrogels was obtained using an x‐ray diffractometer (Malvern Panalytical‐Empyren). The surface elemental composition of the hydrogels was analyzed using x‐ray photoelectron spectroscopy (AXIS Supra+). Solutions of different compositions of hydrogels were loaded into quartz cuvettes against a background of deionized water, and their UV spectral absorption was measured in the 350–600 nm wavelength range using an Agilent Cary 100 UV–vis spectrophotometer.

### 4.3 Mechanical Performance Testing

Tensile stress–strain tests were performed on hydrogels (40 × 10 × 1.5 mm) using an electronic universal testing machine (ZQ‐990LB) at a speed of 80 mm min^−1^. The tensile breaking stress and maximum strain are called breaking strength and breaking elongation, and the toughness of the hydrogel is calculated from the area of the tensile strain–stress curve.

### 4.4 Adhesion Testing

The adhesive strength of the hydrogel was measured using the lap‐shear test, in which the hydrogel was cut into 10 × 40 mm samples and sandwiched between two substrates. A standard lap‐shear test was performed at room temperature at a speed of 10 mm min^−1^, and the bond strength was calculated by dividing the stress by the bond area. The sample size of this experiment was *n* = 3, and the error bars in the corresponding figure represent standard deviation (SD), which indicates the fluctuation of replicate data.

### 4.5 Sensing Performance Testing

Evaluated the sensing performance of hydrogel sensors using an electrochemical workstation (Shanghai Chenhua CHI 760E), using the same strips as those used in mechanical testing. For the sensitivity of tensile sensing performance, the gauge factor (*GF*) is defined by Equation (1):

(1) $$\mathrm{GF}=\frac{\Delta R/R_{0}}{\xi }=\frac{\left(R/R_{0}\right)/R_{0}}{\xi }$$

where ξ represents the deformation magnitude of the hydrogel strip, *R* denotes the real‐time resistance, and *R*_0_ indicates the initial resistance.

For pressure sensing, sensitivity (*S*) is defined by Equation (2):

(2) $$S=\frac{\Delta R/R_{0}}{\Delta P}=\frac{\left(R/R_{0}\right)/R_{0}}{\Delta P}$$

where ∆*P* represents the magnitude of pressure applied to the hydrogel, *R* denotes the real‐time resistance, and *R*_0_ indicates the initial resistance.

### 4.6 Human Body Sensing, Handwriting Sensing, and Morse Code Testing

Copper electrodes were connected to both ends of the hydrogel sensor, and changes in resistance were recorded using a Shanghai Chenhua CHI 760E electrochemical workstation. During testing, the sensor was placed in close contact with the relevant body part or test substrate. The human body sensing experiments involved only the first author; the subject was fully informed and voluntarily signed a written informed consent form. The ethical certificate (Ref—MA2.5D1GF Gel Sensor) was acquired prior to the beginning of these experiments, with a copy of the certificate attached at the end of Supporting Information.

### 4.7 Computational Details

Geometry optimizations were performed using Gaussian 09 [66], employing the B3LYP functional [67] and 6–31G* [68] basis set. SDD pseudopotential and pseudopotential basis sets were used for the transition metal ferric ion. The geometry optimizations were run with dispersion corrections from Grimme's D3 model [69] with Becke‐Johnson damping factors [70]. Harmonic vibrational frequency calculations were performed to confirm the stationary points as true minima, as well as to provide thermodynamic corrections to the SCF energies. The binding energy is defined as E(AB)‐E(A)‐E(B), and the binding energy is calculated using the single‐point energy calculated by the higher‐level method [71]. The ball and stick structures were created using GaussView 6.1.1 [72].

### 4.8 Machine Learning

This experiment was conducted in a Python 3.12 environment for the implementation of machine learning models. In order to realize the collection and processing of signals generated by the hydrogel and send them to the computer, the HC‐06 data receiving module, HC‐05 data transmitting module, voltage regulator, and Arduino microcontroller self‐assembled data transmission module were used for the construction of hardware.

## Author Contributions

H.D., B.B.X., and H.W. developed the concept. H.D., Y.L., C.L., F.Z., and Y.Z. performed experiments and data acquisition. H.D., Y.L., M.M.R., W. T., C.C., H.W., and B.B.X. performed the numerical analysis and FE simulation. All authors participated in the data analysis and the processes of figures and visualizations. The work was supervised by B.B.X. and H.W. The manuscript was written by H.D., Y.L., C.L., M.M.R., B.B.X., and H. W., with contributions from all authors.

## Conflicts of Interest

The authors declare no conflicts of interest.

## Supporting information




**Supporting File 1**: advs77052‐sup‐0001‐SuppMat.docx.

**Supporting File 2**: advs77052‐sup‐0002‐VideoS1.mp4.

**Supporting File 3**: advs77052‐sup‐0003‐VideoS2.mp4.

## Data Availability

The data that supports the findings of this study are available in the supplementary material of this article.
