# Supplementary material for: Robust Stitching Interface and Deep Learning Empowered Hydrogel Human‐Machine Interface
Source: Adv Sci (Weinh). 2026 Aug 6;13(60):e77052. doi: 10.1002/advs.77052 (PMC13445332; doi:10.1002/advs.77052)
Supplement: Supplementary file 1 [file ADVS-13-e77052-s003.docx]

Supporting Information

**Robust Stitching Interface and Deep Learning empowered Hydrogel Human-Machine Interface**

*Hao Dong, Yiming Luo, Chuanliu Liu, Mahamood Modupe Rasheedat, Wenjiayi Tan, Fangyu Zhao, Yixue Zhang, Chris Connor, Hua Hou, Hamdy Khamees Thabet, Ben Bin Xu*, Huige Wei**

Hao Dong, Yiming Luo, Chuanliu Liu, Fangyu Zhao, Yixue Zhang, Huige Wei

State Key Laboratory of Bio-based Fiber Materials, Tianjin Key Laboratory of Multivariate Identification for Port Hazardous Chemical Substances, Tianjin Key Laboratory of Brine Chemical Engineering and Resource Eco-Utilization, College of Chemical Engineering and Materials Science, Tianjin University of Science and Technology, Tianjin 300457, China

Wenjiayi Tan

College of Chemistry Fuzhou University, Fuzhou 350116, China

Mahamood Modupe Rasheedat, Chris, Connor, Ben Bin Xu

School of Engineering, Physics and Mathematics, Northumbria University, Newcastle Upon Tyne NE1 8ST, UK

Hua Hou

School of Materials Science and Engineering, Taiyuan University of Science and Technology, Taiyuan, 030024, China

Hamdy Khamees Thabet

Center for Scientific Research and Entrepreneurship, Northern Border University, Arar 73213, Saudi Arabia

Corresponding Author Email: [ben.xu@northumbria.ac.uk](mailto:ben.xu@northumbria.ac.uk) (B.B.X);

[huigewei@tust.edu.cn](mailto:huigewei@tust.edu.cn) (H.W)

**Table of Contents**

**Supplementary Figures**

**Figure S1.** XPS spectra of Na element in MA_2.5_D_1_GF hydrogel.

**Figure S2.** XPS spectra of C element in MA_0_D_1_GF hydrogel

**Figure S3.** XPS spectra of O element in MA_0_D_1_GF hydrogel.

**Figure S4.** Toughness and strain at the break of the MA*_x_*D*_y_*GF hydrogel.

**Figure S5.** Cyclic stability of the MA_2.5_D_1_GF hydrogel before and after 30 days.

**Figure S6.** Digital photographs of MA_2.5_D_1_GF hydrogel subjected to a load of about 14.7 N.

**Figure S7.** Schematic of adhesion test.

**Figure S8.** Adhesion of MA*_x_*D*_y_*GF hydrogel to PET.

**Figure S9.** Mechanism of the MA_2.5_D_1_GF hydrogel adhesion to PET.

**Figure S10.** Adhesion of MA*_x_*D*_y_*GF hydrogel to PET before and after 30 days.

**Figure S11.** Adhesion of MA_2.5_D_1_GF hydrogel to glass after 10 cycles of cyclic peeling.

**Figure S12.** Adhesion of MA_2.5_D_1_GF hydrogel to wood after 10 cycles of cyclic peeling.

**Figure S13.** Adhesion of MA_2.5_D_1_GF hydrogel to Cu after 10 cycles of cyclic peeling.

**Figure S14.** Adhesion of MA_2.5_D_1_GF hydrogel to NBR after 10 cycles of cyclic peeling.

**Figure S15.** Sensitivity (*S*) of hydrogel sensor at different pressures (0-16kPa).

**Figure S16.** Relative resistance changes of the hydrogel sensor under different pressures (0.1-0.5 kPa).

**Figure S17.** Relative resistance changes in the hydrogel sensor under different strain pressures (1-16 kPa).

**Figure S18.** Comparison of the waveform peaks of the hydrogel sensor under different pressures (1-16 kPa).

**Figure S19.** Changes in relative resistance at different compression frequencies (0.3-2 Hz) under a fixed pressure of 0.5 kPa.

**Figure S20.** Changes in the relative resistance of the hydrogel sensor after 200 compressions at a fixed pressure of 0.5 kPa.

**Figure S21.** Resistance change curves produced by writing characters (A, B, C) individually.

**Figure S22.** Morse code book.

**Figure S23.** Resistance signals bent at 30° and 90°, respectively.

**Figure S24.** MA_2.5_D_1_GF sensor and MA_0_D_1_GF sensors stability testing encapsulated by PET.

**Figure S25.** Model loss function.

**Figure S26.** Accuracy curve.

**Supplementary Tables**

**Table S1.** Composition and content of different hydrogels.

**Table S2.** Comparison of mechanical properties of hydrogel with other reported literature.

**Table S3.** Comparison of strain properties and adhesive properties of hydrogel with other reported literature.

**Table S4.** Comparison of sensing performance of hydrogel sensors with other reported literature.

**Supplementary Video**

**Video S1.** Stretching process of MA_2.5_D_1_GF hydrogel with high strain.

**Video S2.** MA_2.5_D_1_GF sensor in the information transfer process.

**Supplementary Figures**

**Figure S1.** XPS spectra of Na element in MA_2.5_D_1_GF hydrogel.

**Figure S2.** XPS spectra of C element in MA_0_D_1_GF hydrogel.

**Figure S3.** XPS spectra of O elements in MA_0_D_1_GF hydrogel.

**Figure S4.** Toughness and strain at the break of the MA*_x_*D*_y_*GF hydrogel.

**Figure S5.** Cyclic stability of the MA_2.5_D_1_GF hydrogel before and after 30 days.

**Figure S6.** Digital photographs of MA_2.5_D_1_GF hydrogel subjected to a load of about.14.7 N.

**Figure S7.** Schematic of adhesion test.

**Figure S8.** Adhesion of MA*_x_*D*_y_*GF hydrogel to PET.

**Figure S9.** Mechanism of the MA_2.5_D_1_GF hydrogel adhesion to PET.

**Figure S10.** Adhesion of MA*_x_*D*_y_*GF hydrogel to PET before and after 30 days.

**Figure S11.** Adhesion of MA_2.5_D_1_GF hydrogel to glass after 10 cycles of cyclic peeling.

**Figure S12.** Adhesion of MA_2.5_D_1_GF hydrogel to wood after 10 cycles of cyclic peeling.

**Figure S13.** Adhesion of MA_2.5_D_1_GF hydrogel to Cu after 10 cycles of cyclic peeling.

**Figure S14.** Adhesion of MA_2.5_D_1_GF hydrogel to NBR after 10 cycles of cyclic peeling.

**Figure S15.** Sensitivity (*S*) of hydrogel sensor at different pressures (0-16kPa).

**Figure S16.** Relative resistance changes of the hydrogel sensor under different pressures (0.1-0.5 kPa).

**Figure S17.** Relative resistance changes in the hydrogel sensor under different strain pressures (1-16 kPa).

**Figure S18.** Comparison of the waveform peaks of the hydrogel sensor under different pressures (1-16 kPa).

**Figure S19.** Changes in relative resistance at different compression frequencies (0.3-2 Hz) under a fixed pressure of 0.5 kPa.

**Figure S20.** Changes in the relative resistance of the hydrogel sensor after 200 compressions at a fixed pressure of 0.5 kPa.

**Figure S21.** Resistance change curves produced by writing characters (A, B, C) individually.

**Figure S22.** Morse code book.

**Figure S23.** Resistance signals bent at 30° and 90°, respectively.

**Figure S24.** MA_2.5_D_1_GF sensor and MA_0_D_1_GF sensors stability testing encapsulated by PET.

**Figure S25.** Model loss function.

**Figure S26.** Accuracy curve.

**Supplementary Tables**

**Table S1** Composition and content of different hydrogels.

| Hydrogels | AM  (g) | AANa:DMC  (g:g) | Gelatin  (g) | FeCl_3_  (g) | H_2_O  (ml) | MBAA  (mg) | APS  (mg) |
| --- | --- | --- | --- | --- | --- | --- | --- |
| MA_0_D_1_GF | 0.5 | 0:1 | 0.3 | 0.2 | 10 | 6 | 25 |
| MA_1.5_D_1_GF | 0.5 | 1.5:1 | 0.3 | 0.2 | 10 | 6 | 25 |
| MA_2_D_1_GF | 0.5 | 2:1 | 0.3 | 0.2 | 10 | 6 | 25 |
| MA_2.5_D_1_GF | 0.5 | 2.5:1 | 0.3 | 0.2 | 10 | 6 | 25 |
| MA_3_D_1_GF | 0.5 | 3:1 | 0.3 | 0.2 | 10 | 6 | 25 |

**Table S2.** Comparison of mechanical properties of hydrogel with other reported literature.

| **Hydrogels** | **Strain (%)** | **Stress (kPa)** | **Ref.** |
| --- | --- | --- | --- |
| **MA_2.5_D_1_GF** | **2409** | **391.3** | **This work** |
| P(AAm-co-MPC) | 1550 | 54.6 | ^[1]^ |
| PQ_40_C_50_B_1.5_ | 324 | 58.9 | ^[2]^ |
| PAAm-UAHSPC | 714.5 | 83.4 | ^[3]^ |
| PSBMA/OHA-CMCs/GO | 878 | 133 | ^[4]^ |
| PaAAC-Fe^3+^ | 1708.7 | 146.6 | ^[5]^ |
| PAA-SCMC | 1675 | 250 | ^[6]^ |
| PVA-PAA-PDA-DES | 1040 | 25.6 | ^[7]^ |

**Table S3.** Comparison of strain at break and adhesive strength of hydrogel with other reported literature.

| **Hydrogels** | **Strain at break** | **Adhesive strength** | **Ref.** |
| --- | --- | --- | --- |
| **MA_2.5_D_1_GF** | **2409%** | **PET (47.8 kPa)** | **This work** |
| A_9_S_1_CP | 675% | Plastic (11.5 kPa) | ^[8]^ |
| PAM/PVA/LiTFSI_-6%_ | 964% | Plastic (2.75 kPa) | ^[9]^ |
| Polyzwitterion-clay nanocomposite hydrogel | beyond 1500% | PET (about 18 kPa) | ^[10]^ |
| LS-Fe^3+^/CNC/P(AM-APBA-AAS) | 1170% | Plastic (18.8 kPa) | ^[11]^ |
| PAHCT | 990% | PET (38.5 kPa) | ^[12]^ |

**Table S4.** Comparison of sensing performance of hydrogel sensors from other reported literature.

| **Hydrogel Type** | **Gauge Factor (GF)** | **Sensitivity (S)** | **Multimodal Sensors** | **Ref.** |
| --- | --- | --- | --- | --- |
| **MA_2.5_D_1_GF** | **GF_1_ = 13.4 (0-50%)**  **GF_2_ = 8.02 (50-250%)**  **GF_3_ = 5.64 (250-500%)** | **S_1_ = 0.68 kPa^-1^**  **(0-1 kPa)**  **S_2_ = 0.19 kPa^-1^**  **(1-5 kPa)**  **S_3_ = 0.021 kPa^-1^**  **(5-16 kPa)** | **Motion monitoring & Handwriting recognition** | **This work** |
| MHPBA-GO | GF_1_ = 1.8 (0-90%)  GF_2_ = 2.1 (90-160%)  GF_3_ = 8.5 (160-200%) | NA | Motion monitoring | ^[13]^ |
| PTSL | GF = 2.52 (0-680%) | NA | Motion monitoring | ^[14]^ |
| Ox-(Gy/Pz)-Car | GF_1_ = 0.78 (0-100%)  GF_2_ = 2.02 (100-380%)  GF_3_ = 3.66 (380-500%) | S_1_ = 0.04 kPa^-1^  (0-90 kPa)  S_2_ = 0.041 kPa^-1^  (90-120 kPa)  S_3_ = 0.07 kPa^-1^  (120-150 kPa) | Motion monitoring & Handwriting recognition | ^[15]^ |
| P(AAm-THMA-ILs)/PP | GF_1_ = 0.78 (0-200%)  GF_2_ = 2.02 (200-500%)  GF_3_ = 10.64 (500-900%) | S_1_ = 0.052 kPa^-1^  (0-20 kPa)  S_2_ = 0.0053 kPa^-1^  (20-60 kPa)  S_3_ = 0.0011 kPa^-1^  (60-10 kPa) | Motion monitoring & Handwriting recognition | ^[16]^ |
| PATV | GF_1_ = 3.2 (0-200%)  GF_2_ = 6.1 (200-400%)  GF_3_ = 8.7 (400-600%)  GF_4_ = 10.4 (600-800%) | S_1_ = 0.14 kPa^-1^  (0-3 kPa)  S_2_ = 0.05 kPa^-1^  (3-70 kPa)  S_3_ = 0.01 kPa^-1^  (7-16 kPa)  S_4_ = 0.001 kPa^-1^  (16-580 kPa) | Motion monitoring & Handwriting recognition | ^[17]^ |
| PVA-EBPD | GF_1_ = 3.098 (0-40%)  GF_2_ = 5.133 (40-70%)  GF_3_ = 8.294 (70-100%) | NA | Motion monitoring | ^[18]^ |
| PBLG | GF_1_ = 1.74 (0-200%)  GF_2_ = 2.35 (200-400%)  GF_3_ = 3.17 (400-1000%) | NA | Motion monitoring | ^[19]^ |

**Ethical Approval certificate**

**References**

[1] K. He, P. Cai, S. Ji, Z. Tang, Z. Fang, W. Li, J. Yu, J. Su, Y. Luo, F. Zhang, T. Wang, M. Wang, C. Wan, L. Pan, B. Ji, D. Li, X. Chen, *Adv. Mater.* **2023**, 36, 2311255.

[2] Y. Zhao, R. Wu, Y. Hao, Y. Zhao, X. Zhang, H. Liu, W. Zhai, K. Dai, C. Pan, C. Liu, C. Shen, *Adv. Mater.* **2025**, 37, 2507127.

[3] M. Bai, Y. Chen, L. Zhu, Y. Li, T. Ma, Y. Li, M. Qin, W. Wang, Y. Cao, B. Xue, *Nat. Commun.* **2024**, 15, 10569.

[4] H. Fang, X. Wei, Z. Cui, S. He, W. Shao, *Int. J. Biol. Macromol.* **2025**, 286, 138380.

[5] H. Li, C. B. Chng, H. Zheng, M. S. Wu, P. J. D. S. Bartolo, H. J. Qi, Y. J. Tan, K. Zhou, *Advanced Science* **2024**, 11, 2305702

[6] J. Wei, C. Liu, L. Shi, Y. Liu, H. Lu, *Carbohydr. Polym.* **2025**, 350, 122943.

[7] W. Lin, J. Zhang, G. Pan, Y. Lin, D. Chen, J. Lu, J. Li, J. Huang, Z. Li, X. Lin, Y. Sun, G. Yi, Y. Qin, *Adv. Funct. Mater.* **2025**, e09779.

[8] X. Qing, P. Kalidindi, Z. Liu, A. Vananroye, I. Taurino, A. Katsaounis, P. Fardim, *Int. J. Biol. Macromol.* **2025**, 309, 142841.

[9] X. Dong, W. Chen, X. Ge, S. Li, Z. Xing, Q. Zhang, Z.-X. Wang, *Journal of Energy Storage* **2024**, 89, 111793.

[10] G. Gao, F. Yang, F. Zhou, J. He, W. Lu, P. Xiao, H. Yan, C. Pan, T. Chen, Z. L. Wang, *Adv. Mater.* **2020**, 32, 2004290

[11] J. Wang, P. Du, Y.-I. Hsu, H. Uyama, *Chem. Eng. J.* **2024**, 480, 148324.

[12] R. Wu, T. Zhu, Y. Ni, C. Wu, W. Wang, K. Zhao, J. Huang, Y. Lai, *Adv. Funct. Mater.* **2025**, e15120

[13] L. Tian, T. Liu, Y. Jiang, B. He, H. Hao, *Chem. Eng. J.* **2024**, 497, 154890.

[14] L. Cui, W. Wang, J. Zheng, C. Hu, Z. Zhu, B. Liu, *Carbohydr. Polym.* **2024**, 342, 122406.

[15] Z. Wang, L. Xu, W. Liu, Y. Chen, Q. Yang, Z. Tang, H. Tan, N. Li, J. Du, M. Yu, J. Xu, *Int. J. Biol. Macromol.* **2024**, 276, 133802.

[16] W. Wang, P. Guo, X. Liu, M. Chen, J. Li, Z. Hu, G. Li, Q. Chang, K. Shi, X. Wang, K. Lei, *Adv. Funct. Mater.* **2024**, 34, 2316346

[17] S. Han, Y. Hu, J. Wei, S. Li, P. Yang, H. Mi, C. Liu, C. Shen, *Adv. Funct. Mater.* **2024**, 34, 2401607.

[18] Y. Yan, L. Yu, X. Zhang, Q. Han, Z. Yang, X. Lu, J. Wang, H. Xu, Q. Chen, H. Zhao, *Nat. Commun.* **2025**, 16, 839.

[19] Y. Zhang, Y. Wang, Y. Guan, Y. Zhang, *Nat. Commun.* **2022**, 13, 6671.
